# Cyber-infrastructure and epidemic precautionary policy: evidence from China

**DOI:** 10.3389/fpubh.2023.1198928

**Published:** 2023-07-19

**Authors:** Qiuming Gao, Youlong Hu, Zangyi Liao, Lu Yao

**Affiliations:** ^1^Business School, China University of Political Science and Law, Beijing, China; ^2^School of Political Science and Public Administration, China University of Political Science and Law, Beijing, China

**Keywords:** precautionary policies, cyber infrastructure, epidemic, COVID-19, China

## Abstract

**Introduction:**

The application of technology supported by cyber infrastructure has emerged as a critical factor influencing city management. This study aims to investigate whether the development of cyber infrastructure can enhance cities’ confidence in responding to potential epidemic threats in the context of COVID-19.

**Methods:**

China serves as a good example for both COVID-19 management and smart city construction. We take advantage of a special time point, the 2022 Chinese New Year, to observe cities’ precautionary epidemic policies. We utilize choice models and data from 188 Chinese cities to examine the impact of internet coverage on the degree of policy relaxation.

**Results:**

We found that cities with higher internet coverage tend to adopt looser policies. In the benchmark regression, for every 1 percentage point increase in internet coverage, the likelihood of implementing loose measures increases by 0.9 percentage points. This result remains robust across different classifications of policies. We also addressed potential endogeneity issues by using the instrumental variables method.

**Discussion:**

Our study indicates that effective management of epidemics in the modern era requires not only the utilization of traditional medical resources but also the incorporation of new city features, such as information technology infrastructure.

## 1. Introduction

Public health events caused by epidemics have been plaguing and accompanying global social development (1). Since the enactment of the International Health Regulations in 2007, the World Health Organization has declared seven Public Health Emergencies of International Concern, including the COVID-19 pandemic. As a result, precautionary policy-making has become a significant topic in the field of public health. With the improvement of targeted vaccines and drugs, research attention has gradually shifted from focusing on the COVID-19 virus to understanding how we can learn from the experiences of epidemic management in order to effectively address future threats. Large-scale public health events are characterized by the rapid spread and strong pathogenicity of the viruses, whose impact extends beyond the health system. Consequently, responding to such events can hardly rely on medical measures alone, but necessitates the implementation of non-medical interventions and the synergy of the various economic and social resources behind them. In this context, how the development of city infrastructure may affect the decisions on precautionary policies, has become a matter of concern.

Each outbreak of a pandemic urges lessons to be learned. Existing literature has predominantly focused on intervention policies (2–4). For the precautionary or preventive measures, attention has been given to the institutional mechanism. For instance, based on the observation of SARS outbreak, Smith noted that non-medical interventions can affect public risk perception which leads to severe economic consequences, and thus management mechanisms on risk perception need to be established in advance (5). In the context of the H1N1 pandemic, Baekkeskov compared vaccination approaches between the Netherlands and Denmark. He found that since decisions about public health events are usually faced with urgent decision-making under uncertainty, it was crucial to pre-establish relevant public health management norms (6). The Ebola outbreak in West Africa emphasized the urgent need for establishing emergency mechanisms in both the local healthcare system and international aid efforts (7, 8). These studies address the importance of preset management mechanisms, but the factors and local endowments that determine the eventual adoption of such mechanisms have not been fully explored.

An important factor influencing city development in recent years has been the application of information and intelligence technology supported by internet infrastructure (9). Just as it has had a profound impact on the industrial sector, information and intelligence technology has also played a significant role in urban governance. This impact can be seen not only in enhancing the quantity and efficiency of public service delivery, reducing administrative procedures and time costs, but more importantly, in helping governments collect and analyze large amounts of data for more accurate and scientific policy-making (10–12). Data-driven policy making is increasingly valued by governments and has been proved to play a role in a wide range of areas (13). Furthermore, the connection on the internet has also led to changes in the way social resources are coordinated. The establishment of digital platforms enables information sharing between government agencies and citizens as well as external stakeholders, providing diverse and interactive channels of communication and integration between parties (14, 15).

**Figure 1:**
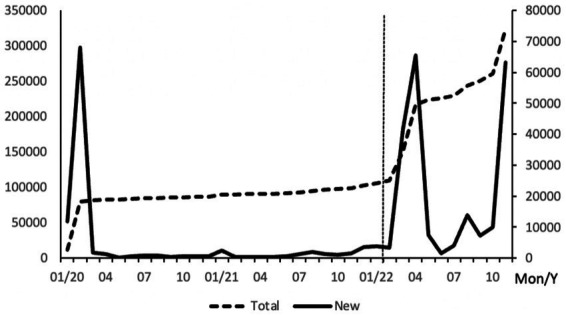
New COVID-19 cases in China monthly from Jan 2020 to Nov 2022. National Health Commission of China. data summed from 31 provinces and province-level municipalities.

**Figure 2:**
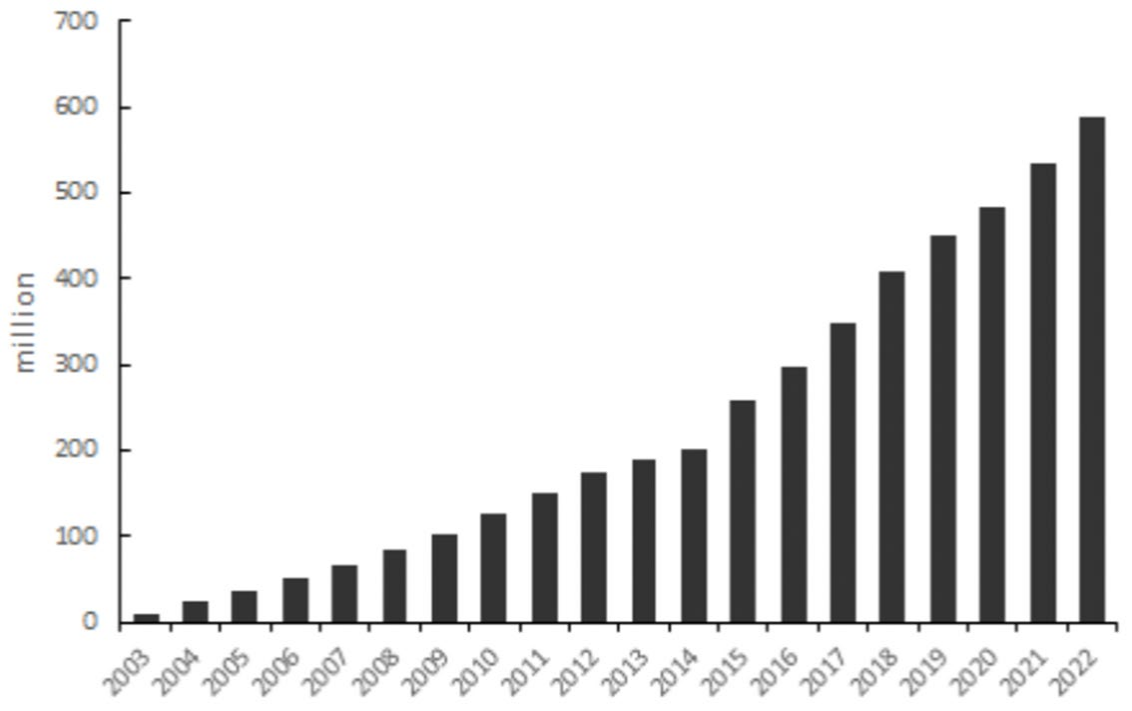
Growth in Internet users in China since 2003. Statistical Bulletin of National Economic and Social Development, Statistical Bulletin of China’s Communication Industry Development.

So does the improvement of cyber infrastructure also help to increase the confidence of cities in dealing with potential epidemic outbreaks? China serves as a good example to observe this issue. China adopted a strict management policy aiming at achieving zero cases during the COVID-19 epidemic, which allowed the country to maintain a very low number of infections during the prevalence of the Delta variant (Figure 1). During this period, the primary concern for most city governments was not how to implement medical or non-medical interventions, but rather how to take precautions against potentially possible outbreaks. The preventive measures adopted by each city reflected their local adaptation based on city characteristics. At the same time, cities in China are also actively engaged in smart city construction (16). Internet coverage, a major indicator of cyber infrastructure, has experienced rapid growth in recent years (Figure 2). Information and intelligence technology applications have permeated various aspects of municipal management (17). In the management of COVID-19 confirmed cases and risk-exposed populations, new technologies merge as a powerful addition to the epidemic management toolbox and are used to provide broad social coordination support, which differs from the situation during the SARS period in 2003 (18). In this context, it presents an opportunity for us to empirically test the relationship between cyber infrastructure and the cities’ precautionary policy choices.

This paper examines the impact of internet infrastructure on COVID-19 preventive policy choices based on data from 188 cities in China. We take advantage of a special time point, the 2022 Chinese New Year (vertical line in Figure 1), when large-scale population movement was anticipated. We identify policy differences based on the measures implemented prior to the movement. We found that cities with higher internet coverage tend to adopt looser preventive measures. This result is robust across different classifications of management measures. To address possible endogeneity problems, we employ the instrumental variables method and include additional control variables, and the conclusion remains unchanged. Our study shows that the epidemic management in the new era relies not only on traditional medical resources but also on new city features such as information technology conditions. The importance of the latter may even surpass that of certain medical infrastructure, such as hospital beds.

The possible contributions of this paper are as follows: first, it verifies the role of cyber infrastructure in the formulation of precautionary policies in the context of COVID-19, thus adding to the lessons learnt from COVID-19 prevention and control. We address the significance of non-medical technology factor asides from that of the traditional health care facilities. Second, it presents new findings on the influencing factors for epidemic preventive policy-making, which has direct policy implications. Third, our research provides new empirical evidence for the impact of smart city construction in the field of public health, thereby supplementing the existing literature on urban studies.

The rest of this paper is organized as follows: section two provides the background and sorts out the ways in which information and intelligence technology plays a role in epidemic management; Section three describes the research methodology and data; Section four reports the empirical results and includes discussions on robustness and endogeneity. Finally, section five concludes.

## 2. Background

### 2.1. China’s COVID-19 management measures

Some studies have divided the development stages of the COVID-19 pandemic in China from different time points and perspectives (19, 20). From the perspective of preventive and control measures, China’s anti-epidemic policy can be divided into three stages. The period before March 2020 is the Emergency phase. In the face of the sudden outbreak of the epidemic, this stage aimed to swiftly exterminate the virus in its early stages. Rigorous and comprehensive control measures were implemented such as the lockdown of key cities and the nationwide shutdown of work and production. These measures aimed to sever all possible routes of human-to-human and human-to-object transmission by temporarily suspending economic and social activities (21). Due to the limited knowledge of the new virus’s transmission in the initial stage, the control measures during this stage were of a very high standard and highly coordinated nationwide. However, these measures came at the cost of substantial loss of economic and social activities and were therefore taken as short-term interim approaches to explore the effective way of managing a new virus (19). The second stage is the Regular management stage from April 2020 to December 2022. With an improved control system and increased vaccine coverage, the objective of this phase was to establish a mechanism for epidemic prevention and control that could minimize disruptions to economic and social development. The focus shifted from rapid eradication to prevention. This stage emphasizes a science-based approach and targeted measures. On the one hand, when cases occurred, the regions and populations that required control measures were gradually refined and narrowed down, making them more precise. On the other hand, monitoring and prevention methods, mainly based on nucleic acid testing, were promoted to replace the control measures implemented after cases arose, helping to balance between economic and social development and epidemic management. Our analysis of epidemic prevention policies is mainly based on this stage. After December 2022, since the dominant epidemic strain transitioned from the previous Delta variant to the new Omicron variant, which is characterized by strong infectivity but weak pathogenicity, control measures and large-scale monitoring measures previously set were replaced in this third stage.

In the formulation of COVID-19 prevention and control system in China, the central authority and local authorities perform respective duties. The *Joint Prevention and Control Mechanism of the State Council* (JPCM), established by the State Council on January 20, 2020, led by the National Health Commission and composed of 32 ministries and commissions, is at the core to coordinate the national epidemic prevention and control efforts. It issues national guidelines for epidemic management measures. The decisions are made based on the *Protocol on Prevention and Control of COVID-19* issued by the National Health Commission. This medical technical guidebook has been updated ten versions by the end of 2022 and provides definitions, treatment methods, and management criteria for infected and high risk-exposed populations. According to the medical protocol and combined with the experience found in practice, JPCM provides guidance at the nation level on the management of key population groups, locations and organizations. It also establishes binding rules that must be followed by all local regions, such as specifying the exact quarantine measures and duration for close contacts with the virus. Local authorities at the provincial, municipal and county levels are responsible for their local epidemic prevention and control respectively, setting localized measures under the requirements of the central authorities and bearing the responsibility for any local epidemic outbreak (22). During the Emergency stage, almost all local authorities adopted rigorous control measures out of prudence, resulting in a relatively uniform approach nationwide, while in the Regular stage, their measures showed greater variation. The JPCM also encourages policy localization with local characteristics. It identified the best practices initiated by individual local communities and promoted them as common standards nationwide. For instance, the health QR code, which was widely used across China during the epidemic, was first introduced by Hangzhou city in Zhejiang province.

A key aspect of epidemic control is to sever the transmission routes. In addition to hospitalizing infected individuals in designated treatment centers, differentiated management measures are imposed on different at-risk groups. These measures mainly include the following: *Centralized quarantine*, which refers to staying in a designated quarantine facility with on-site medical staff assisting in daily health and psychological monitoring as well as conducting regular nucleic acid tests. *Home quarantine*, which involves isolation in a separate house or room within the individual’s residence. It requires regular health monitoring and door-to-door nucleic acid testing or antigen self-testing, the results of which need to be reported to the specialized management team of the community through the internet. People under home quarantine are not allowed to leave their residences or receive visits from outsiders. *Home health monitoring* is also conducted within the individual’s residence, but with more relaxed requirements for contact with co-inhabitants. It does not mandate isolation in a single room. Individuals under home monitoring are permitted to go out when necessary. Table 1 provides a summary of these measures. Vulnerable people are categorized into close contacts, close contacts of close contacts, and personnel exposed to epidemic-related premises. Each group is subject to differentiated measures. For instance, close contacts are required to undergo centralized quarantine, while close contacts of close contacts only receive home quarantine. The specific measure for each group is not constant but is modified according to updates in the medical technical protocol, resulting in variations during different time periods. For example, close contacts were initially required to undergo 14 days of centralized quarantine, which was subsequently reduced to 7 days in June 2022.

**Table 1:** Measures for risk populations.

*Measure*	*Contents*
Centralized quarantine	Stay in a designated place determined by local authorities, where on-site medical staff are available. Undergo daily morning and evening health monitoring, psychological monitoring, and regular nucleic acid testing in accordance with epidemic prevention regulations. During this period, leaving the premises is prohibited, and all visits are denied.
Home quarantine	Quarantine at a personal residence, in a separate house or separate room. Undergo health monitoring, door-to-door nucleic acid tests or antigen self-testing in accordance with regulations. A specialized team from the community conducts online information collection and provides on-site management assistance. Going out is prohibited and all visits are denied.
Home health monitoring	Quarantine at a personal residence, preferably in a separate house or room. Undergo health monitoring, door-to-door nucleic acid tests or antigen self-testing in accordance with regulations. A specialized team from the community conducts online information collection and provides on-site management assistance. Going out is allowed when necessary.

**Figure 3:**
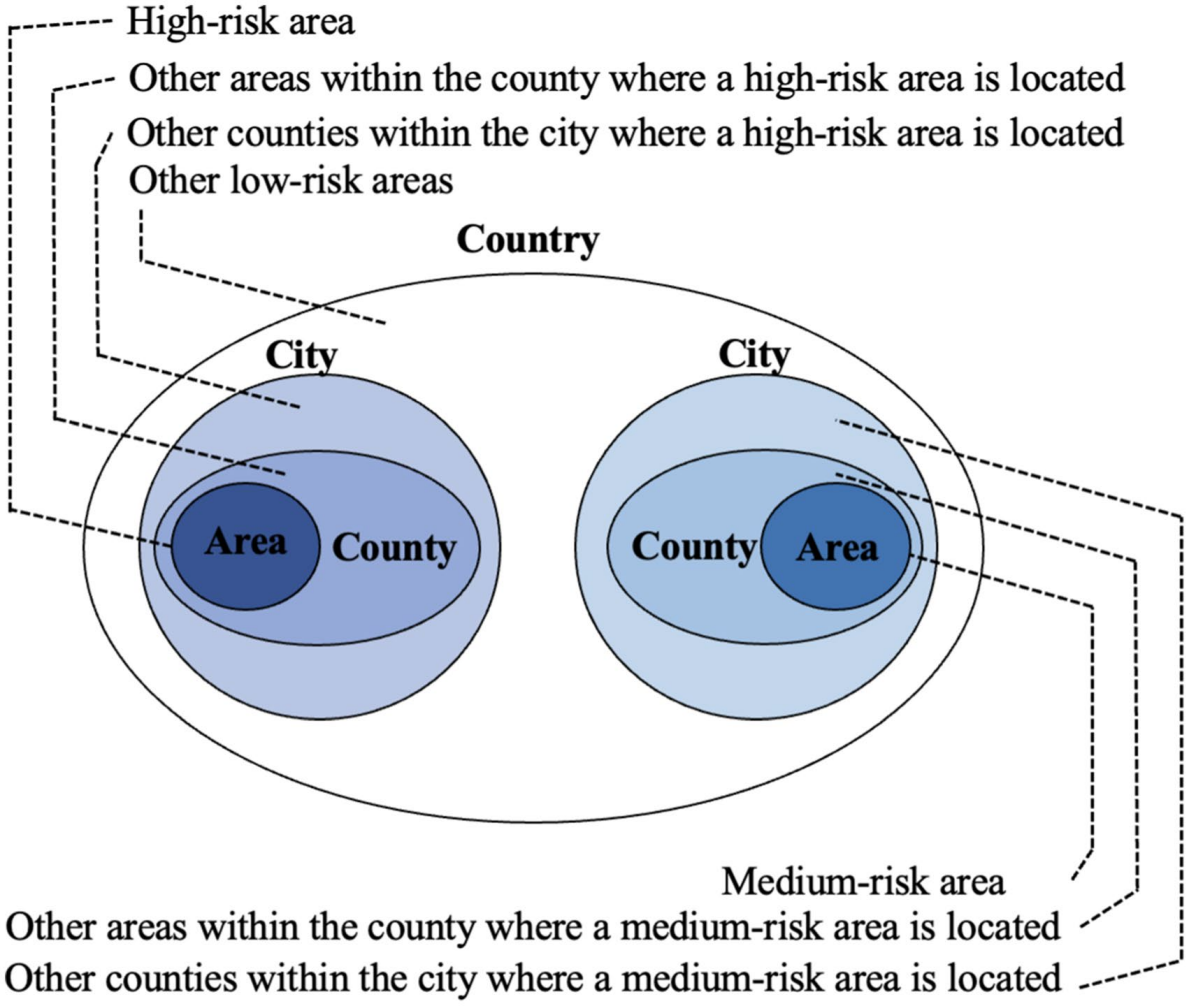
Risk Categories.

In addition to controlling the populations at risk within a specific region, it is equally important to cut off potential cross-regional transmission routes as part of the management policy. According to the JPCM, regions are classified into three risk levels based on a comprehensive evaluation of factors such as local population and the number of infected cases within a given period. These risk levels include high-risk, medium-risk and low-risk areas. Correspondingly, regions at each level adopt differentiated control measures. The region (area) was initially defined as the county level or above during the emergency stage, but gradually contracted to towns, blocks, and later building units in the regular management stage (In November 2022, the medium-risk category was abolished). For the cross-regional travel, strict restrictions are imposed by JPCM for the outflow from high- and medium-risk areas. Accordingly, local authorities at the destination usually impose control measures on these people. For people coming from low-risk areas, further distinctions are made considering the proximity to medium- or high-risk regions, as there is still a possibility of exposure to the epidemic. For instance, JPCM in its *Work Plan for the Prevention and Control of COVID-19 during the New Year and Chinese New Year in 2022*, singles out two types of low-risk areas, namely “other areas within the county where a medium- or high-risk area is located” and “other counties within the city where a medium- or high-risk area is located “(Figure 3 illustrates the classification of risk categories). For the former, in practice the management measures for people living in these areas are typically the same as those in the corresponding medium- or high-risk area, and thus require “strict restrictions on travel (across provinces)” as specified by the *Work Plan*, while for the latter, “no (cross-province) travel unless necessary” is required. Both areas are subject to measures distinct from those applied to general low-risk areas. Local authorities also follow these guidelines to further classify the population and arrange control measures. Table 2 summarizes these cross-regional mobility control requirements. It is important to note that there are no uniform regulations for the management of individuals coming from areas close to high- or medium-risk areas. Instead, the approach depends on the discretion of local governments. This perspective allows us to identify differences in the design of prevention and control policies among different cities.

**Table 2:** Measures for cross-regional mobility of people (issued in December 2021).

*Risk category of the source region*		*Control guidelines of JPCM*	*Measures in the destination made by*
High-risk area		Strict restrictions on (inter-provincial) travel	Local authorities
Medium-risk area		Strict restrictions on (inter-provincial) travel	Local authorities
Low-risk area	Other areas within the county where a high-risk area is located	Strict restrictions on (inter-provincial) travel	Local authorities
	Other areas within the county where a medium-risk area is located		
	Other counties within the city where a high-risk area is located	No (across-province) travel unless necessary	Local authorities
	Other counties within the city where a medium-risk area is located		
	Other low-risk areas	None	Local authorities

### 2.2. The role of internet and information technology in China’s epidemic control

As new tools for urban governance, the internet and information technologies have played a broad role in epidemic control during COVID-19. It can be summarized into the following four aspects.

*Epidemiological investigation.* The tracing of cases and identification of contacts have transitioned from the traditional manual approach to locating them with cell phone signals. The holders of mobile phones that presented at the time and geographical range of risk exposure can be quickly reached by CDC personnel and their risk level can be categoried automatically. This greatly improves the efficiency of epidemiological investigations. Based on the traceability of mobile phones, China has employed QR code in epidemic management with authorization from the public. Each person is assigned a *Health QR Code* displayed through a mobile phone program. The code’s color (green, yellow, or red) corresponds to the individual’s risk level, which is calculated based on their public visits. The code is used to access public venues. The form and functions of the codes vary across cities, determined by the municipal authorities. Some are integrated with additional features such as displaying virus test results and vaccination status, as well as binding with electronic transportation cards for automatic identification on public transport. In addition to the health QR codes for intra-city use, for cross-city travel, the China Academy of Information and Communication Technology in collaboration with China’s three major mobile communication operators, has established *Digital Travel Records*. These records identify and track the mobile phone users’ paths through base stations, making it easy for people to check whether they have recently passed through risk areas. This system helps filter people at risk and avoids misreporting of travel information. As of June 2022, digital trip records have served 1.6 billion cell phone users in China, processing over 55.6 billion inquiries.

*Medical treatment capacity.* Internet-based remote diagnosis and treatment has become a powerful complement to on-site hospital services. Firstly, online platforms that support video consultations and surgical assistance break the previous geographical limitations of medical facilities, thereby expanding the emergency mobilization capability to deal with large-scale outbreaks (23). Secondly, quarantine and control measures may consume limited medical resources, reducing medical accessibility for common and chronic diseases (24). However, the demand for medical services tends to rise during epidemics as people become more sensitive to their health conditions due to the presence of cases around them. In response to these medical needs, online medical services provided by internet healthcare companies and hospital internet programs have developed rapidly during the COVID-19 epidemic in China (25). The availability of higher internet coverage enables these online services to reach a wider population, thereby enhancing primary care capacity during the epidemic.

*Information dissemination.* Emerging infectious diseases often lack effective means of prevention and treatment, which tends to cause public panic when they break out (26). In China during the COVID-19 outbreak, nearly all city governments launched special epidemic prevention and control sections on their official websites and developed dedicated mobile APP or program modules. This approach serves two purposes. Firstly, it facilitates the transparent dissemination of information regarding the progression of the epidemic, enabling citizens to receive timely alerts about changes in the risk status of each region. Secondly, these platforms efficiently delivers new knowledge about the virus and the corresponding updates on protective measures to the public. These information platforms provide the authoritative interpretations of management policies and the knowledge about disease prevention, thus guiding the public to a scientific understanding of the epidemic, raising public awareness, and preventing public panic.

*Urban governance and resource coordination.* At the city level, nearly all cities have established electronic coordinating systems for COVID-19 prevention and control. These systems utilize technology support from the internet, internet of things, and big data computing to integrate functions such as contacts tracing, reporting confirmed case, epidemic monitoring and analysis, and emergency command and dispatch. This replaces the traditional manual communication methods with a more timely and efficient approach, promoting information sharing and collaboration across government departments and agencies (27). At the community level, residential communities are organized into “grid” for management. For instance, Longgang District in Shenzhen city divided its population of 4.3 million into 3,823 community-based blocks. Each community block has dedicated staff responsible for monitoring residents’ risk conditions and managing those under control measures. Much of their work is conducted through internet or mobile tools such as Wechat, a widely used chatting APP in China, for communication with local residents. For the communities under access control due to the epidemic, technology tools are also employed to provide daily necessities. Household needs are reported through the internet, and digital files are created to track special needs. Some communities even established online platforms to connect with external suppliers, so as to match the residents’ demands timely and precisely with available supplies.

## 3. Methodology

### 3.1. Empirical strategy

We use the management measures targeting cross-city movement during the Chinese New Year in 2022 to identify differences in cities’ precautionary epidemic policies. The Chinese New Year is the most important festival in Chinese culture, during which people return to their hometowns for family reunions, making it the most mobile period of the year. In 2019, the number of passengers (including trains, buses, passenger ships and airplanes) reached 421 million, equivalent to 30% of the national population (21). The massive influx of people poses a potential risk of epidemic spread. In response to this challenge, the JPCM of the State Council issued the *Work Plan for the Prevention and Control of COVID-19 during the New Year and Chinese New Year in 2022*, based on which local authorities also issued their specific measures. In 2022, the Chinese New Year fell on February 1st (indicated by the vertical line in Figure 1). Prior to this date, the epidemic situation in China was generally stable. On January 29th, among the 188 cities included in our sample, the city with the highest number of current cases was Anyang in Henan Province, with 400 cases, while 165 cities had no existing cases. Only three cities reported new cases on that day, with the largest number being 22 in Hangzhou, Zhejiang Province. This suggests that at that time, cities did not have a heavy burden of controlling the epidemic outbreak, but still faced the potential threat of sporadic cases. Therefore, policy-making at that time reflected precautionary considerations to deal with the potential risk.

Using the Chinese New Year as the focal point of our study also offers several advantages. Firstly, during this time, very few cities are immune to the potential impacts of epidemic risks. The movement of people across the country, as individuals return to their hometowns, is difficult to predict, unlike population movements driven by commercial activities. Secondly, as workers return home, factories typically reduce production intensity or even shut down for approximately 3 weeks during the New Year period. This alleviates concerns on the economic consequences of the epidemic in policy-making, compared to other times of the year. Thirdly, almost all cities update their policies in preparation for the New Year population flow, making it easier for us to make comparison. If we were to choose a random time point, cities may have varying policies due to individual needs, leading to potential biases in our analysis. The specific date we use for policy extraction is January 29, 2022, which was the last working day before the Chinese New Year’s day.

Specifically, our regression model can be represented as follows:


$$y_{c}=\alpha +\beta Cyber_{c}+X_{c}\gamma +\varepsilon_{c}$$

where $y_{c}$ indicates the strict degree of the precautionary epidemic policy in city c.$Cyber_{c}$ measures the city’s cyber infrastructure. $X_{c}$ is a set of control variables for city characteristics.

In constructing the dependent variable, we focus on the city’s management policy specifically targeting people travelling from “other areas within the county where a medium-risk area is located.” This level represents the lowest level that requires control measures according to the JPCM. Areas above this level, i.e., the medium- and high-risk areas, as well as other areas within the county where a high-risk area is located, are associated with a high risk of exposure. Local authorities at the destination typically adopt strict control measures for people arriving from these areas, leading to policy convergence. Conversely, below this level, local authorities tend to impose no control measures, resulting in similar practices. Thus, the diversification at this level helps capture policy differences among cities. The specific measures implemented for people falling within this category include (for cities that impose a combination of more than one measures, only that of the highest degree was taken) 14 days of centralized quarantine, 7 days of centralized quarantine, 3 days of centralized quarantine, 14 days of home quarantine, 7 days of home quarantine, 3 days of home quarantine, 14 days of home monitoring, 3 days of home monitoring, and self-monitoring (i.e., no control measures). Recognizing that centralized quarantine requires individuals to stay in designated places for isolation, which significantly impacts their daily lives, we further classified these measures into a dummy variable indicating centralized or non-centralized management, where $y_{1}=0$ denotes centralized management including centralized quarantine for 14 days, 7 days or 3 days, and $y_{1}=1$ for other cases including all types of home management and no measures. We use larger values to indicate looser measures. This variable serves as the dependent variable in our benchmark regression. In the robustness section, we utilize alternative classifications to validate our results. Additionally, we employ choice models alongside LPM to reflect the features of different dependent variables.

The independent variable, cyber infrastructure, is represented by the city’s internet coverage following existing literature (28). It is calculated as: Internet coverage = number of internet broadband access users/year-end population *100.

Control variables include local healthcare resources and other city characteristics. Healthcare resources are represented by the number of hospital beds per thousand population and the number of doctors per thousand, which measure the city’s medical infrastructure from the perspectives of hardware and software, respectively. Moreover, as most of the epidemic-controlling activities are led by the local authorities and thus funded through local public finance, we also incorporate the city’s fiscal revenue *per capita* as a proxy for the fiscal space for health^1^. Other city characteristics include city size (population), level of socio-economic development (GDP *per capita*, *China Integrated City Index* ranking), and degree of urbanization (proportion of urban population, population density). Considering the possible demonstration effect of provincial capital cities, a dummy variable of provincial capital city is also added. Fiscal revenue *per capita*, population, population density and GDP *per capita* are in logarithmic form in regressions.

### 3.2. Data

Data on the precautionary policies of cities were collected from a manual search on the official websites of local authorities and from official media reports on the internet. City characteristics were obtained from the *China City Statistical Yearbook*. CICI ranking was sourced from *Yunhe City Research Institute*. In order to mitigate the endogeneity problem, we used data from the year prior to the outbreak of COVID-19, i.e., the year of 2019, as the epidemic itself may influence social and economic performance.

The original dataset contains all cities in the Yearbook. We exclude those of special status to form our regression sample. Cities excluded are: cities from ethnic autonomous regions, as well as Qinghai, Yunnan, and Guizhou provinces, which are classified as ethnic regions by the Ministry of Finance for public finance funding purposes; cities from provinces that are adjacent to other countries; province-level municipalities directly under the Central Government; and Hebei province, which was preparing to host the Winter Olympics. Additionally, eight cities that could not obtain the specific policies were also excluded, as well as six cities with missing variables in the Yearbook. Our final sample contains 188 cities.

Table 3 provides descriptive statistics. It can be seen that there is a significant difference in internet coverage between the two city groups, with a difference of more than 10 percentage points. Cities with loose policies also tend to exhibit a higher number of doctors per thousand population, higher fiscal revenue *per capita*, higher GDP *per capita*, a larger proportion of urban population and a higher CICI ranking.

**Table 3:** Summary statistics.

	Full (*N* = 188)		$y_{1}=0$ (*N* = 117)		$y_{1}=1$ (*N* = 71)	
	Mean	*sd*	Mean	*sd*	Mean	*sd*
Internet coverage (%)	34.443	*16.747*	30.380	*12.70*	41.138	*20.212*
Number of hospital beds (/1,000)	4.895	*1.611*	4.910	*1.385*	4.871	*1.937*
Number of doctors (/1,000)	2.673	*1.067*	2.484	*0.781*	2.984	*1.369*
Fiscal revenue *per capita* (yuan)	5610.82	*6670.87*	4093.42	*3176.35*	8111.31	*9590.12*
Population (10,000)	477.777	*264.612*	472.667	*280.637*	486.197	*237.540*
GDP *per capita* (yuan)	66610.4	*36855.1*	56918.4	*27642.8*	82581.8	*44120.2*
CICI ranking	142.931	*84.629*	161.595	*76.487*	112.437	*88.877*
Proportion of urban population (%)	37.604	*22.500*	35.550	*20.367*	40.990	*25.424*
Population density(10,000/km^2^)	0.052	*0.037*	0.045	*0.026*	0.064	*0.049*
Provincial capital	0.080	*0.272*	0.068	*0.253*	0.099	*0.300*

## 4. Empirical results

### 4.1. Basic results

Table 4 shows the regression results. Column 1 includes only internet coverage as a right-hand variable. It can be seen that the coefficient of internet coverage is significantly positive, indicating that cities with better internet infrastructure tend to adopt looser precautionary policies. Column 2 introduces the number of hospital beds and the number of doctors as controls, which represent local medical resources. The coefficient of internet coverage is still significantly positive, with the scale nearly unchanged. Internet facilities function independently of the traditional medical infrastructure. Column 3 further introduces city characteristics, and the coefficient of internet coverage remains stable, suggesting that cyber infrastructure does have a positive effect on cities’ confidence in responding to potential epidemic risks. In column 3, for every 1 percentage point increase in internet coverage, the probability of a city adopting loose measures increases by 0.8 percentage points.

**Table 4:** Basic results.

	(1)	(2)	(3)	(4)	(5)
	LPM	LPM	LPM	Logit	Probit
Dependent var.	$y_{1}$	$y_{1}$	$y_{1}$	$y_{1}$	$y_{1}$
Internet coverage	0.009^***^	0.009^**^	0.008^**^	0.008^*^	0.008^**^
	(0.002)	(0.004)	(0.004)	(0.004)	(0.003)
Hospital beds		−0.185^***^	−0.189^***^	−0.205^***^	−0.208^***^
		(0.030)	(0.034)	(0.036)	(0.035)
Doctors		0.163^**^	0.173^**^	0.205***	0.211^***^
		(0.074)	(0.076)	(0.078)	(0.076)
Fiscal revenue *per capita* (ln)		0.109	0.034	0.048	0.054
		(0.074)	(0.099)	(0.100)	(0.099)
Population (ln)			−0.041	−0.038	−0.036
			(0.083)	(0.074)	(0.076)
GDP *per capita* (ln)			0.107	0.079	0.080
			(0.139)	(0.134)	(0.137)
CICI ranking			−0.000	−0.000	0.000
			(0.001)	(0.001)	(0.001)
Urban proportion			−0.002	−0.002	−0.002
			(0.003)	(0.002)	(0.002)
Population density (ln)			0.016	0.023	0.020
			(0.058)	(0.058)	(0.057)
Provincial capital			0.130	0.136	0.129
			(0.166)	(0.172)	(0.166)
Constant	0.065	−0.353	−0.480		
	(0.077)	(0.497)	(1.481)		
*N*	188	188	188	188	188
R-sq	0.098	0.237	0.251		

[1] ^***^, ^**^, and ^*^ represent statistical significance at the 1, 5, and 10% levels, respectively. [2] Robust standard errors are reported in the parentheses. [3] Columns 4 and 5 report marginal effects.

Since the dependent variable is in discrete form, we also change the LPM setting and use binary choice models for regression. Columns 4 and 5 of Table 4 show the results of Logit regression and Probit regression, respectively. As can be seen, the marginal effects of internet coverage are consistent with that in LPM, showing that the positive effect of network infrastructure is not affected by the model setup.

Among the control variables, it is noteworthy that the number of hospital beds and the number of doctors act in a different way. Although the number of doctors has significantly positive effect on cities’ choosing loose policies, the effect of hospital beds is negative. This suggests that increase in physical facilities alone does not directly lead to a boost in confidence in epidemic prevention and control; even in the case of medical resources, the confidence depends on the corresponding capability building. This supports the idea that software improvements on cities’ epidemic responding capability may play an important role in precautionary decision making.

### 4.2. Robustness

#### 4.2.1. Alternative classifications of precautionary policies

In our benchmark regression, cities’ management measures are grouped into centralized management and non-centralized management to construct the dependent variable $y_{1}$. In this section we use other alternative classification methods to check the robustness of our findings. These classifications are shown in Table 5. On the basis of $y_{1}$, $y_{2}$ categorizes the measures into three groups, with $y_{2}=1$ for centralized management defined the same as in $y_{1}$, $y_{2}=2$ for home management including home quarantine and home monitoring, and $y_{2}=3$ for no measures. On the basis of $y_{2}$, $y_{3}$ further subdivides centralized management into centralized management for 14 days ($y_{3}=1$) and centralized management for 7 days and below ($y_{3}=2$), thus forming a variable containing four categories. We use $y_{2}$ and $y_{3}$ to replace $y_{1}$ as dependent variable, respectively, and repeat regression. Considering dependent variables take values with the ordinal feature, Ordered Probit models are used. The regression results are shown in columns 1 and 2 of Table 6. For each additional 1% increase in internet coverage, in column 1 the probability of choosing centralized management, home management and no control measures will increase by −0.6, 0.1, and 0.5%, respectively, while in the setting of column 2, the probability of choosing 14-day centralized management, 7-day centralized management or below, home management, and no control measures will increase by −0.4%, nearly 0, 0.1, and 0.3%, respectively. These results indicate that higher internet coverage is associated with looser management measure, which is consistent with our benchmark finding.

**Table 5:** Classifications of management measures.

y	Definitions
$y_{1}$	$y_{1}=0$ for centralized management, $y_{1}=1$ for non-centralized management
$y_{2}$	$y_{2}=1\;$ for centralized management, $y_{2}=2$ for home management, $y_{2}=3$ for no control measures
$y_{3}$	$y_{3}=1$ for 14-day centralized management, $y_{3}=2$ for 7-day centralized management or below, $y_{3}=3$ for home management, $y_{3}=4$ for no control measures
$y_{4}$	$y_{4}=0$ for strict measure group, $y_{4}=1$ for loose measure group

**Table 6:** Robust checks.

		(1)	(2)	(3)	(4)
		Oprobit	Oprobit	Probit	Probit
Dependent var.		$y_{2}$	$y_{3}$	$y_{4}$	$y_{1}$
Internet coverage				0.016^*^	
				(0.010)	
	y=1	−0.006^**^	−0.004^*^		
		(0.003)	(0.002)		
	y=2	0.001^*^	−0.000		
		(0.001)	(0.000)		
	y=3	0.005^**^	0.001^*^		
		(0.002)	(0.001)		
	y=4		0.003^*^		
			(0.002)		
Mobile phone coverage					0.008^***^
					(0.002)
Province FE		No	No	Yes	No
*N*		188	188	80	188

[1] ^***^, ^**^, and ^*^ represent statistical significance at the 1, 5, and 10% levels, respectively. [2] Other control variables include population (ln), GDP per capita (ln), proportion of urban population, population density (ln), CICI ranking (not in column 3), hospital beds per thousand population, doctors per thousand population, fiscal revenue per capita (ln) and provincial capital city dummy. [3] Robust standard errors are reported in the parentheses. [4] All columns report marginal effects.

Policy-making in cities can be influenced by the opinions of their provinces. Some provinces have even unified the policies over their cities, which makes the policy choice of these cities unable to reflect their own characteristics. We then control for provinces and categorize the policy leniency of cities by comparison within provinces. We exclude those provinces with completely uniform policies and take only those provinces with differed policies within its boundary, and divide the cities into strict group and loose group based on comparison with other cities within the province. The grouping method follows the rationale of the previous classifications. Specifically, if within the province cities take either centralized or non-centralized management measures for our targeted people flow, those cities with centralized management are grouped as being strict while those with non-centralized management being loose; if all the cities within the province take non-centralized measures, those with home management are sent to the strict group, while those with no measures to the loose group; if all the cities within the province take centralized measures, those with centralized management for 14 days are considered being strict, and those for 7 days and below are considered being loose. If the measures for our targeted people flow (i.e., travelling from “other areas within the county where a medium-risk area is located”) are completely unified within the province, the policies for “other counties within the city where a county with medium-risk areas is located” is taken and used to divide cities following the above method. In this way we obtain binary variable $y_{4}$, which we use to replace the dependent variable in the benchmark model, while controlling for province fixed effects (as the cities’ CICI rankings lead to multicollinearity concern with province fixed effect, we exclude CICI ranking from this regression). The results are shown in column 3 of Table 6. It can be seen that the coefficient of internet coverage remains positive and significant, and our results remain robust to this classification.

#### 4.2.2. Mobile phone coverage

The support of cyber infrastructure for epidemic prevention and control is not solely generated by broadband internet; mobile network also makes its contribution. Therefore, we also use mobile phone coverage as an alternative measure of cyber infrastructure, and put it into the regression. The mobile phone coverage of the city is calculated as: mobile phone coverage = number of mobile phone subscribers at year-end /total population at year-end *100. Data is from the China City Statistical Yearbook. Column 4 of Table 6 provides the results of the repeated regression. It can be seen that the coefficient of mobile phone coverage is positive and significant, which echoes our benchmark finding.

### 4.3. Endogeneity

Decisions on precautionary policies may not be entirely based on objective conditions, but also involve subjective factors of the authorities such as cautious or bold city governance styles (22), which may also affect the development of information infrastructure. Omitting these variables can lead to endogeneity issues. To alleviate this concern, we use two methods to verify our findings.

First, we use the instrumental variable method. The geographic distance from each city to Hangzhou was used as an instrument. Hangzhou is among the most developed cities in China in terms of digital economy. The health QR code widely used during COVID-19 period in China was originally invented by Hangzhou, and then promoted to its local province and the whole country. In Chinese literature, the geographic distance to Hangzhou is commonly used as an instrument to represent the level of digital economy development of the city (29). We use IV-Probit regression based on this instrumental variable, and the results are shown in columns 1 to 2 of Table 7. Column 1 shows the results of the first-stage regression with internet coverage as the dependent variable. It can be seen that the coefficient of distance is negative and significant, indicating that greater distance to Hangzhou is associated with lower internet coverage of the city, which suggests that distance is a good instrument. Column 2 shows the second-stage results. The coefficient of internet coverage is still positive and significant, which corroborates the findings of the benchmark regression. This suggests that the effect of internet coverage is independent of the effect of subjective factors.

**Table 7:** Endogeneity analysis.

	(1)	(2)	(3)	(4)	(5)	(6)
	IV-Probit		Probit	Probit	Probit	Probit
Dependent var.	Internet coverage	$y_{1}$	$y_{1}$	$y_{1}$	$y_{1}$	$y_{1}$
Internet coverage		0.099^***^	0.008^**^	0.008^**^	0.006	0.002
		(0.008)	(0.003)	(0.003)	(0.004)	(0.004)
Distance to Hangzhou (ln)	−2.632^*^					
	(1.579)					
Current outbreak			0.114	−0.068		
			(0.105)	(0.237)		
Internet coverage ^*^ Current outbreak				0.005		
				(0.006)		
Past outbreaks					−0.297^***^	−0.568^***^
					(0.070)	(0.137)
Internet coverage ^*^ Past outbreaks						0.009^**^
						(0.004)
*N*	187	187	188	188	176	176

[1] ^***^, ^**^, and ^*^ represent statistical significance at the 1, 5, and 10% levels, respectively. [2] Other control variables include population (ln), GDP per capita (ln), proportion of urban population, population density (ln), CICI ranking (not in column 3), hospital beds per thousand population, doctors per thousand population, fiscal revenue per capita (ln) and provincial capital city dummy. [3] Robust standard errors are reported in the parentheses. [4] Probit regressions report marginal effects. [5] Cities in Hubei Province are excluded from regressions in Column 5 and 6 as outliers.

Second, we add on control variables that may contribute to the formation of subjective styles of cities. In the context of epidemic management, we use two variables indicating the city’s COVID-19 experience, namely the severity of the city’s current outbreak and the severity of its past outbreaks. The former is measured by a dummy variable which takes a value of 1 if the number of current existing cases is above zero and 0 otherwise. The latter is measured by a dummy indicating “whether the city’s cumulative number of cases is high “, which takes a value of 1 if the city’s cumulative number of cases exceeds the median and 0 otherwise. Case data are obtained from the Baidu website epidemic statistics column as of January 28, 2022. Columns 3 and 4 of Table 7 show the regression results with the current outbreak variable and its cross-term with internet coverage added in controls. It can be seen that the coefficients of both the variable and its cross-term are not significant, and their inclusion has little effect on the coefficient of internet coverage. This suggests that the ongoing outbreak does not affect precautionary policy making of cities. Columns 5 and 6 of Table 7 show the regression results after adding the past outbreak variable and its cross-term. Cities in Hubei Province are excluded from this regression as outliers considering their extraordinarily large case numbers compared with other Chinese cities since the epidemic initially broke out in that province. It can be seen from column 5 of Table 7, that although past outbreaks are supposed to increase a city’s experience in managing epidemics, they actually decrease the probability of a city taking loose precautionary policy, indicating that these cities become more cautious. The coefficient of internet coverage tends to remain positive but insignificant. A closer look in column 6 including the cross-term shows that the coefficient of the cross-term is positive and significant. Thus internet infrastructure is able to moderate the cautiousness of experiencing past outbreaks, thereby helping to offset its effect. These results confirm our findings that information infrastructure does contribute to the relaxation of precautionary epidemic policies.

## 5. Conclusion

We examined the precautionary epidemic policies of 188 cities in China in the face of COVID-19 and found that cities with higher levels of internet coverage tended to develop looser policies when faced with unknown risk exposures. This result remains robust across different policy classifications. Our study shows that cyber infrastructure gives confidence in cities’ public health management, a role that may exceed that of the traditional medical infrastructure such as hospital beds. Therefore, to prepare for future pandemics, cities need to look beyond the healthcare system and prioritize the application of technology to establish comprehensive capabilities for epidemic response.

Our study confirms that cyber infrastructure, as a new foundation for development, also plays a role in the health sector. As described in the background section, the internet-enabled techniques applied to epidemiological investigation, treatment capability expansion and dissemination of epidemic knowledge, feature in ultra-high analytical speed and efficiency, which enable them to achieve effective epidemic control at the early stage of an outbreak. This greatly transforms the epidemic management practices from the traditional manual approach. The effectiveness of this new model gives cities the confidence to respond to epidemics, which further translates into the confidence to adopt loose precautionary policies that entails less interference to social activities in the face of an epidemic threat. This correlation is underpinned by the fact that a larger network coverage corresponds to a greater abundance of technological management tools and a superior new management model. Consequently, the adoption of looser precautionary policies is not a subjective decision, but rather a consequence of objective, superior cyber-infrastructure conditions.

Our paper also suggests that the smart city development can help empower cities to respond to public health emergencies. A limitation of our study is that we only focus on China. Different countries may have distinct social mores and cultures that can influence policy decisions, suggesting that the factors influencing policy-making may vary. Nevertheless, the management of epidemics in all countries should be grounded in certain objective conditions. Therefore, the findings of this paper hold instructive value even for countries with different socio-economic backgrounds. Specifically, we have focused on the role of networks as city infrastructure rather than the specific applications. Future research could further investigate the role of cyber-infrastructure in different countries and against different public health events.

## Data availability statement

The original contributions presented in the study are included in the article/supplementary material, further inquiries can be directed to the corresponding author.

## Author contributions

QG led the overall study. She designed the study, led the data collection and analysis, and wrote the manuscript. YH contributed to the data analysis and literature review, as well as assist in manuscript writing. ZL contributed to the study design, reviewed the manuscript and put inputs in the writing of the first manuscript. LY contributed to the data interpretation and manuscript edits. All authors contributed to the article and approved the submitted version.

## Funding

This paper was funded by Program for Young Innovative Research Team in China University of Political Science and Law.

## Conflict of interest

The authors declare that the research was conducted in the absence of any commercial or financial relationships that could be construed as a potential conflict of interest.

## Publisher’s note

All claims expressed in this article are solely those of the authors and do not necessarily represent those of their affiliated organizations, or those of the publisher, the editors and the reviewers. Any product that may be evaluated in this article, or claim that may be made by its manufacturer, is not guaranteed or endorsed by the publisher.

## Footnotes

^1^We thank the reviewer for pointing this out.
